# Load-Dependent Performance of Repair Techniques for Corrosion-Induced Pinhole Defects in Agricultural Pipelines

**DOI:** 10.3390/ma19153182

**Published:** 2026-07-25

**Authors:** Jae-Hwan Lee, Sooho Kim, Chan-Gi Park, Hyun-Oh Shin, Nemkumar Banthia

**Affiliations:** 1 Department of Agricultural and Rural Engineering, Chungnam University, Daejeon 34134, Republic of Korea; dlwoghks033@o.cnu.ac.kr (J.-H.L.); ksh.kimsooho@gmail.com (S.K.); 2 Research and Development Centre, Construction Material Test Laboratory Co., Ltd., Eumseong-gun 27734, Republic of Korea; 3 Department of Regional Construction Engineering, Kongju National University, Yesan 32439, Republic of Korea; 4 Department of Civil Engineering, The University of British Columbia, Vancouver, BC V6T 1Z4, Canada; banthia@civil.ubc.ca

**Keywords:** thin-walled steel pipes, pinhole damage, pipeline rehabilitation, GFRP composite, multi-joint clamp, overlay welding, four-point bending, tensile performance

## Abstract

**Highlights:**

- The proposed repair method for corroded steel pipelines was experimentally evaluated.
- Localized corrosion significantly reduced the structural capacity of pipelines.
- The proposed repair method effectively restored the load-carrying capacity.
- The proposed method can reduce replacement costs and service interruptions.

**Abstract:**

Agricultural steel pipelines are essential components of pressurized irrigation systems, yet localized corrosion-induced pinholes severely compromise their structural integrity by creating critical stress concentrations. To address the lack of performance-based maintenance guidelines, this study experimentally evaluates three repair techniques—a multi-joint hinge clamp, a GFRP composite sleeve, and overlay welding—applied to steel pipes containing simulated pinhole defects representing 6% and 10% circumferential damage. Four-point bending and uniaxial tensile tests were conducted to simulate transverse overburden and longitudinal axial loading encountered in buried pipelines. Results reveal that repair effectiveness strongly depends on both loading mode and damage severity. At 6% damage, all methods effectively restored bending capacity, with the GFRP sleeve achieving near-complete recovery. Under tensile loading, however, external-confinement methods provided limited ductility improvement because they lack a direct axial load-transfer path. In contrast, overlay welding consistently achieved substantial structural restoration by eliminating stress concentrations and shifting fracture to the parent pipe material. Furthermore, a significant transition in repair performance was observed near the 6% damage level, beyond which confinement-based repairs exhibited reduced efficacy. These findings demonstrate that repair performance cannot be reliably assessed from bending behavior alone and highlight the importance of considering both loading conditions and damage severity in rehabilitation design. The study provides a quantitative framework for load-specific pipeline rehabilitation strategies.

## 1. Introduction

Agricultural water supply networks have progressively transitioned to pressurized steel pipelines; however, these buried, thin-walled systems are inherently vulnerable to aggressive underground environments, frequently suffering from severe pitting corrosion and localized pinhole damage [1,2,3,4,5,6]. From a structural mechanics perspective, such geometric discontinuities act as critical stress concentrators. A localized pinhole exponentially degrades the effective section modulus, rendering the pipe highly susceptible to premature yielding under transverse bending (driven by soil overburden and traffic loads) and catastrophic bursting under longitudinal axial/hoop tension [7,8,9,10,11,12,13,14]. Because bending and tensile loadings impose fundamentally distinct stress demands, the structural adequacy of any localized repair intervention must be rigorously verified under both loading regimes.

To restore the structural capacity of deteriorated pipelines and mitigate leakage, various retrofitting techniques—including direct overlay welding, mechanical multi-joint clamps, and fiber-reinforced polymer (FRP) composite wrapping—have been widely adopted in practice [15]. Although these methods pursue the same objective, they rely on fundamentally different stress-transfer mechanisms and therefore exhibit distinct structural behaviors and failure modes. Overlay welding restores load continuity but introduces heat-affected zones (HAZ) susceptible to localized stress concentration and secondary yielding [16]. Mechanical clamps transfer loads through frictional confinement, making their performance highly dependent on bolt preload and interface conditions and potentially vulnerable to longitudinal slippage under axial loading [17]. In contrast, FRP composites rely on adhesive load transfer, rendering them susceptible to interfacial debonding, particularly in through-wall defects where substrate continuity is lost [18].

Despite these critical mechanistic differences, existing literature has predominantly evaluated these repairs in isolation or exclusively focused on static bending performance. There is a profound lack of comparative studies systematically investigating the distinct failure modes—such as secondary yielding in welds, frictional slip in clamps, or delamination in FRPs—under multiaxial loading conditions. Without such mechanistic cross-validation, the selection of repair techniques remains entirely empirical. Current maintenance practices and guidelines, such as the KDS 67 25 90:2018 (Korean Design Standard for Agricultural Pipeline Maintenance) [19], mandate repair interventions but fail to provide load-specific, performance-based criteria. Applying external-confinement repairs based solely on empirical flexural data may lead to an overestimation of their effectiveness, potentially resulting in inadequate load transfer, interfacial failure, or defect propagation under unexpected pressure transients or ground settlements.

To address this critical knowledge gap and provide robust engineering criteria, this study experimentally investigates the structural performance and failure mechanisms of small-diameter steel pipelines repaired with fundamentally distinct techniques. To quantitatively evaluate the variations in repair efficacy across different degradation states, circular pinholes corresponding to 6% and 10% of the pipe circumference were introduced, simulating early-stage and advanced deterioration, respectively. The structural recovery was evaluated under two distinct loading conditions: four-point bending tests to assess transverse flexural resistance against soil overburden and dynamic vehicular traffic, and uniaxial tensile tests to evaluate longitudinal load-path restoration against thermal contraction and differential ground settlement. The specific interventions investigated include a hinge-type multi-joint clamp, glass fiber-reinforced polymer (GFRP) sleeves, and overlay welding. By systematically comparing the ultimate capacity, ductility, and dominant failure modes, this study aims to replace empirical guesswork with empirical evidence, providing a robust framework for the optimal selection of rehabilitation strategies in aging pipeline networks.

## 2. Materials and Specimens

### 2.1. Material Properties of Thin-Walled Steel Pipes

The experimental program was conducted using carbon steel pipes conforming to KS D 3507 (Korean Standard for Carbon Steel Pipes for Ordinary Piping) [20], which represents the standard pipe material widely employed in domestic agricultural pressurized water supply networks. Although large-diameter pipes (typically ranging from 100 to several hundred millimeters in diameter) are commonly used in agricultural irrigation networks, a scaled-down nominal pipe size of 25A (the KS designation equivalent to DN25; outer diameter: 34.0 mm, wall thickness: 3.25 mm) was deliberately selected for this comparative experimental program. The selected specimens preserved the thin-walled geometric characteristics of field-installed pipelines (D/t ≈ 10.5) while remaining compatible with the load capacity of the testing equipment. This configuration enabled controlled laboratory evaluation of localized stress concentration and repair mechanisms associated with corrosion-induced pinhole defects. As the primary objective of this study was to compare the relative structural effectiveness of different repair methods rather than to reproduce the absolute capacity of a specific pipeline size, the selected geometry provided a practical and representative basis for comparative assessment prior to field-scale application. Nevertheless, scale-dependent effects should be considered when extending the results to full-scale pipeline systems. In particular, variations in the D/t ratio, repair length, and circumferential stiffness may influence the load-transfer behavior and stress distribution around the repaired region. Therefore, the results obtained from the present specimens are primarily intended for comparative evaluation of repair effectiveness and failure mechanisms, while further validation using full-scale systems is required.

To precisely characterize the inherent mechanical properties of the steel pipes—independent of the manufacturer’s general certification—tensile coupon specimens were extracted directly from the pipe walls. To prevent the microstructural alterations and residual stresses typically induced by thermal cutting, a precision waterjet cutting process was employed. The coupon geometry was strictly machined in accordance with ASTM E8 and KS B 0802 standards [21,22]. Three replicate specimens were tested under a controlled crosshead displacement rate of 0.5 mm/min using a 200 kN capacity Universal Testing Machine (Instron 4485, Instron Corporation, Norwood, MA, USA). As summarized in Table 1, the measured properties exhibited excellent agreement with the manufacturer’s mill certificate. The stress–strain response demonstrated a well-defined yield plateau followed by substantial strain hardening, culminating in an elongation at fracture of 50%. This pronounced ductility confirms the material’s suitability for evaluating highly localized stress redistributions during the structural repair tests. Furthermore, the chemical composition strictly complied with KS D 3507 limits (Table 2).

### 2.2. Damage Simulation: Pinhole Defects

In-service degradation of buried steel pipelines frequently manifests as highly localized wall thinning and pitting, ultimately leading to pinhole penetration [23]. To parametrically and reproducibly simulate this severe localized damage, circular through-holes were mechanically drilled at the midspan of the extreme tension fiber (the lower pipe wall under bending) using a precision drill press. While actual pitting corrosion in practical field conditions exhibits irregular geometries and gradual thickness variations, such natural defects introduce significant stochastic variability that can mask underlying structural behaviors. By utilizing precise circular through-holes, the inherent variability of naturally corroded specimens was eliminated, thereby successfully isolating the influence of damage severity and specific repair techniques under identical geometric conditions.

**Figure 1:**
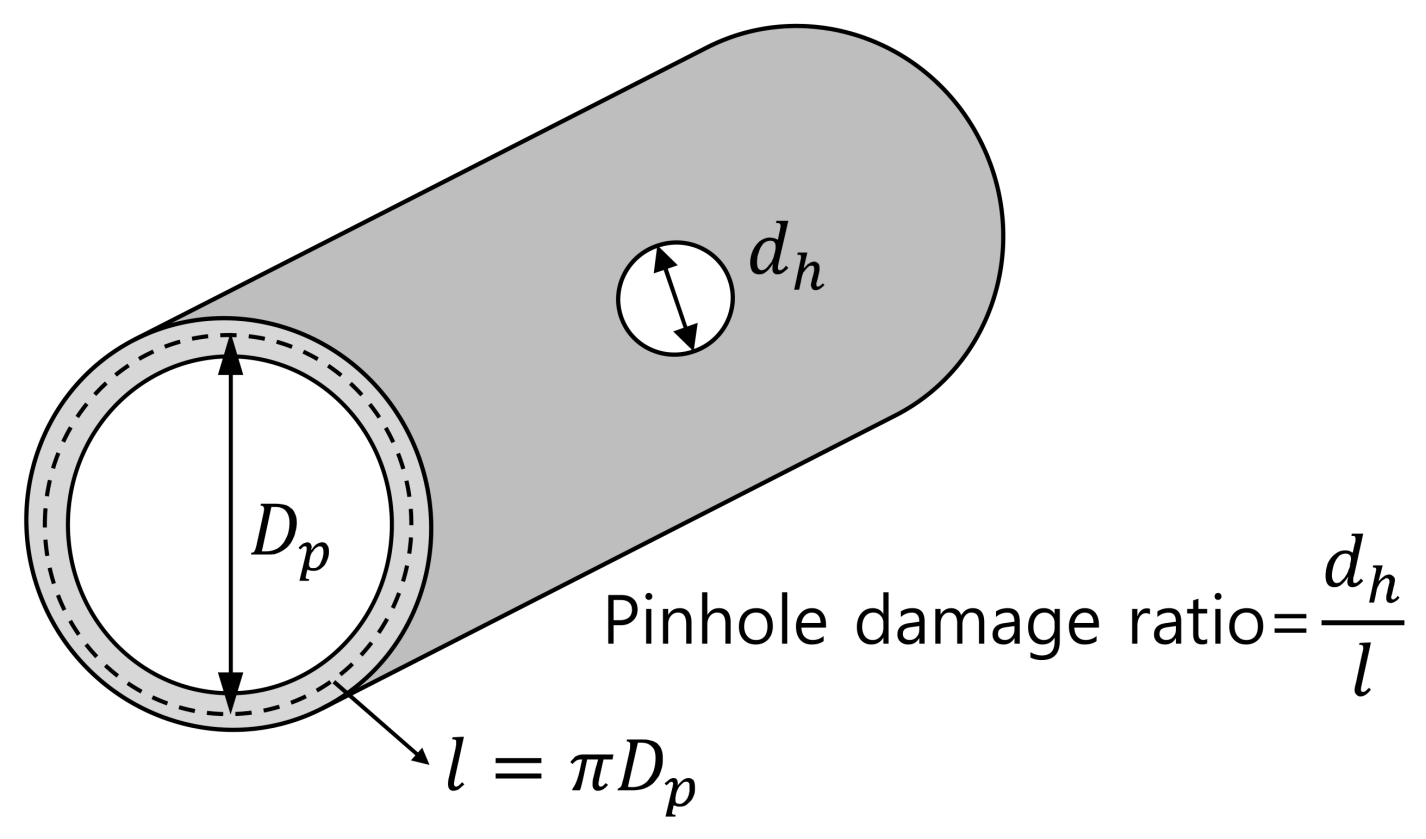
Definition of pinhole damage ratio.

As depicted in Figure 1, damage severity was defined by the ratio of the pinhole diameter (*d_h_*) to the nominal pipe circumference (*l* = *πD_p_*). Two damage levels—6% and 10%—were selected to represent early-stage and advanced deterioration, respectively. This geometric discontinuity not only induces an abrupt reduction in the cross-sectional area but also acts as a severe stress concentrator, thereby enabling the assessment of the conditions under which specific stress-transfer mechanisms of different repair techniques begin to degrade. Notably, no external protective coating or internal lining was applied to the specimens. This controlled condition was intentionally established to isolate the intrinsic structural performance and interfacial behavior of the repair materials, eliminating the confounding variables associated with existing field coatings.

It should be acknowledged, however, that these idealized circular through-holes do not capture the progressive wall thinning and complex stress concentrations associated with actual corrosion pits. Because these geometric discrepancies can alter localized stress-transfer mechanisms near the defect boundary, investigating more realistic corrosion morphologies under complex stress states—coupled with experimentally validated numerical analyses—represents a critical direction for future research.

### 2.3. Repair Methodologies and Stress Transfer Mechanisms

**Figure 2:**
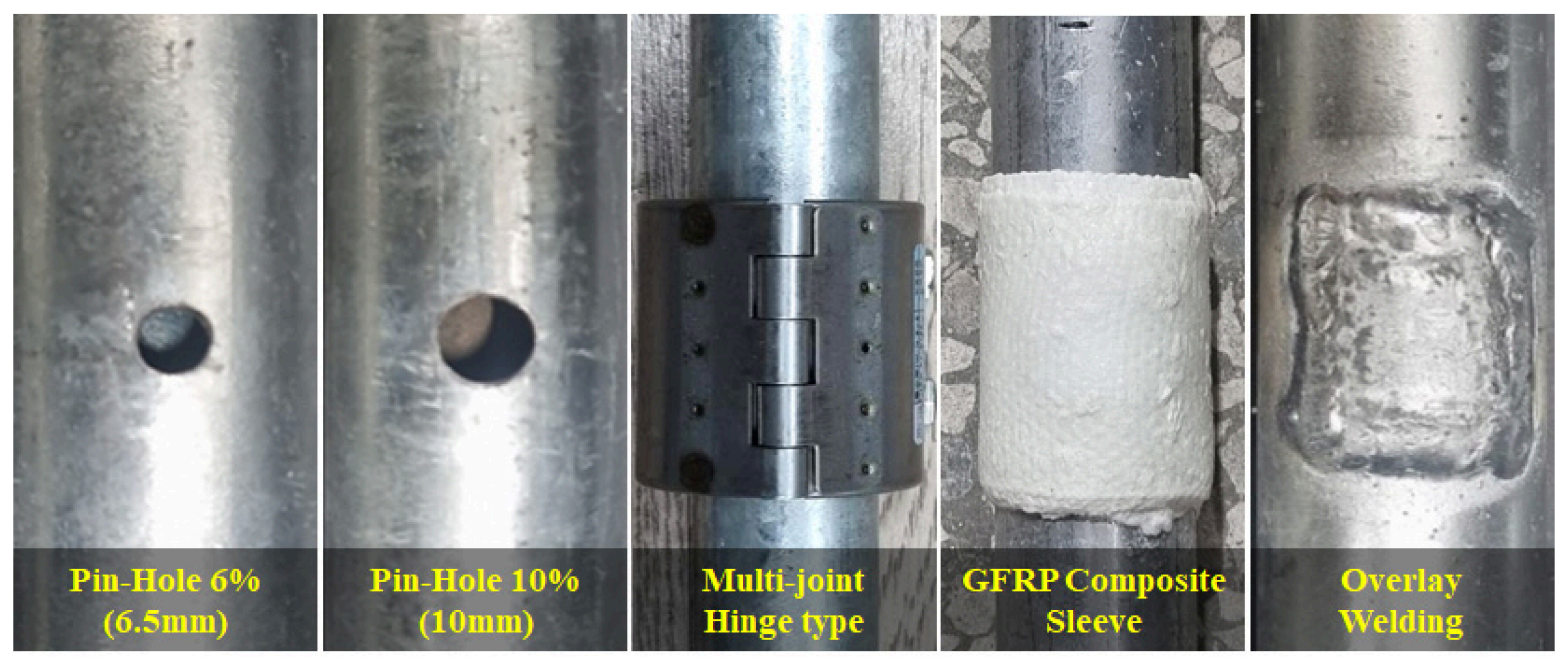
Damaged specimen (6%, 10% damage) and repair methods.

As illustrated in Figure 2, three specific repair configurations were systematically evaluated, selected to represent both emergency and permanent rehabilitation strategies currently practiced or under consideration for steel pipeline systems: (1) multi-joint hinge-type clamp; (2) glass fiber-reinforced polymer (GFRP) composite sleeve; (3) overlay welding. These methods were purposefully selected to represent three fundamentally distinct structural retrofitting paradigms currently employed in pipeline rehabilitation: semi-rigid mechanical confinement, conformal interfacial adhesion, and rigid metallurgical continuity.

#### 2.3.1. Mechanical Coupling: Multi-Joint Clamps (Frictional Stress Transfer)

A multi-joint mechanical clamp represents a rapid-response, emergency intervention primarily reliant on external frictional confinement and compressive sealing. A hinge-type configuration was tested, employing a single-axis hinge for rapid on-pipe rotation. Rather than restoring cross-sectional continuity, this device encapsulates the defect zone and transfers stress through radial compression and interface friction. The clamp body incorporates an internal sealing element (e.g., elastomeric or composite sleeve) to prevent leakage. The geometrical and mechanical specifications of the clamp are detailed in Table 3.

#### 2.3.2. Glass Fiber-Reinforced Polymer (GFRP) Composite Sleeve (Adhesive Stress Transfer)

A glass fiber-reinforced polymer (GFRP) sleeve was employed to evaluate semi-permanent repairs reliant on composite action and interfacial shear transfer. The system comprised a customized glass fiber fabric tape pre-impregnated with a water-activated polyurethane resin. Prior to wrapping, an epoxy putty filler was applied directly over the pinhole to prevent resin infiltration and smooth the stress flow. The GFRP sleeve was circumferentially wrapped over a 57 mm bond length centered on the defect. This technique offers significant field advantages—requiring no heavy machinery and accommodating various pipe materials—by effectively redistributing stress concentrations around the pinhole via the adhesive bond layer. The mechanical properties of the composite system are provided in Table 4.

#### 2.3.3. Overlay Welding (Metallurgical Stress Transfer)

Overlay welding was investigated as a permanent, rigid repair strategy. A steel patch plate of equivalent grade (KS D 3507 [20]) and matching wall thickness (3.25 mm) was fillet-welded directly over the defect region. Unlike clamps or FRP, welding restores full metallurgical continuity and the original section modulus at the defect location. The welded patch was applied over an identical 57 mm central zone to ensure a consistent repair length across all experimental groups, allowing for a direct comparison of structural recovery. While structurally robust, this method inherently introduces a heat-affected zone (HAZ) and requires specialized equipment, representing the upper bound of structural rigidity in this comparative study.

### 2.4. Experimental Matrix and Specimen Designation

A comprehensive experimental matrix comprising nine distinct specimen series was designed, as shown in Table 5. To ensure robust statistical reliability and capture the inherent variability of failure modes, three identical replicates were fabricated and tested for each series, resulting in a total of 27 specimens per loading condition (i.e., 27 for bending, 27 for tension). Specimen designations follow a systematic alphanumeric convention: the prefix ‘H’ denotes a damaged (holed) pipe, the subsequent numeral (6 or 10) indicates the damage severity ratio (%), and the final suffix identifies the applied repair technique (e.g., W for Welding, GF for FRP, MH for Clamp-Hinge). To provide a clearer conceptual understanding of how these different repair configurations restore structural integrity, the underlying reinforcing mechanisms, load transfer pathways, and primary failure modes for each technique are systematically summarized in Table 6.

## 3. Test Setup and Evaluation Metrics

As established in Section 2, in-service steel pipelines are subjected to complex multiaxial demands. To systematically capture the repair effectiveness, two fundamentally distinct loading conditions were evaluated: (1) severe longitudinal axial tension induced by thermal contraction and differential ground movement, and (2) transverse bending stress induced by pipe self-weight, soil overburden, and dynamic vehicle loads acting on buried sections. It should be noted that the experimental program and results presented in this study are limited to quasi-static bending and tensile loading conditions and therefore should not be directly extrapolated to the structural performance under dynamic, cyclic, or fatigue loading.

To rigorously evaluate the structural recovery and distinct stress-transfer mechanisms under both scenarios, independent four-point bending and uniaxial tensile tests were conducted using a 200 kN capacity Universal Testing Machine (Instron 4485). All tests were performed at room temperature (25 ± 2 °C) under displacement control with a constant crosshead displacement rate of 0.5 mm/min. For each experimental series, three replicate specimens were tested to ensure statistical reliability, and the averaged load–displacement responses were utilized for comparative analysis.

### 3.1. Four-Point Bending Test Configuration

Bending specimens were fabricated with an overall length of 500 mm. A clear span of 300 mm was established between the two support rollers, divided equally into three 100 mm segments. Load was applied at the one-third and two-thirds span positions, creating a 100 mm constant-moment region at the midspan where shear forces are theoretically zero. The simulated pinhole defect and all corresponding repair materials were strictly centered within this constant-moment zone to evaluate their performance under the most critical pure flexural stress state.

**Figure 3:**
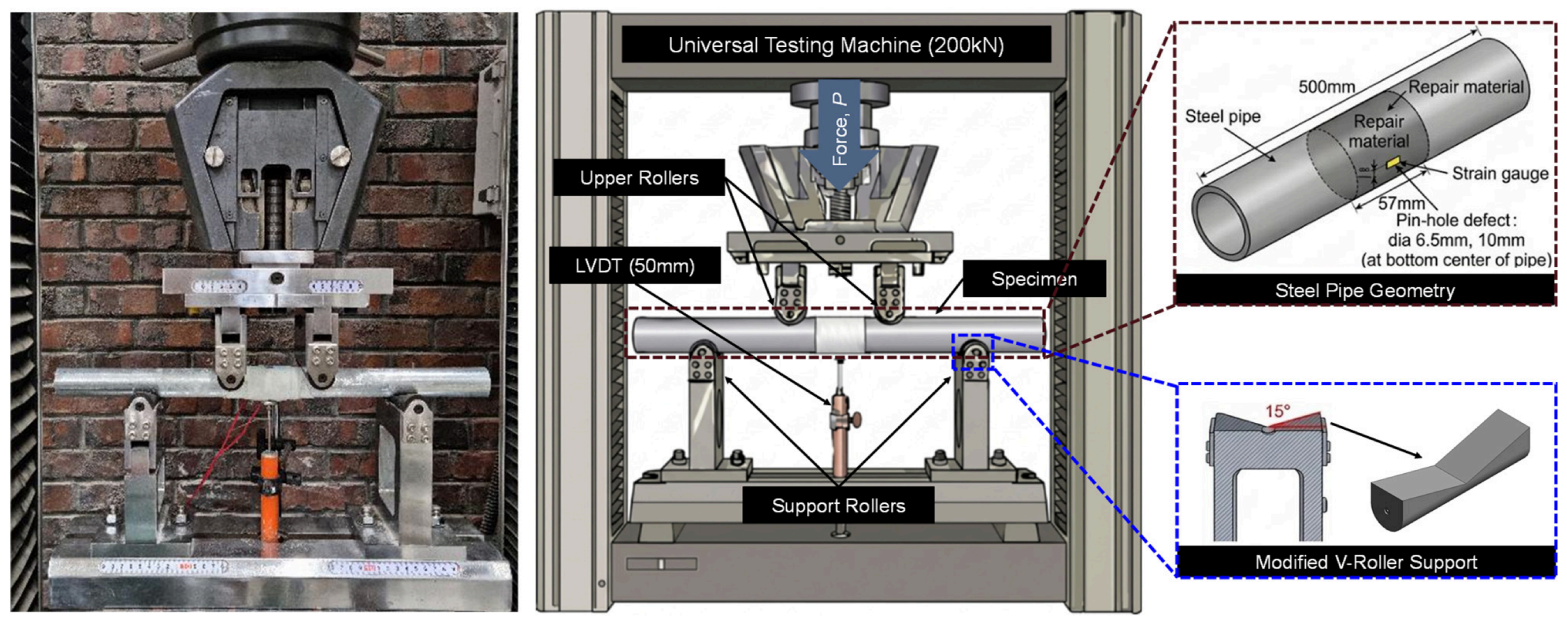
Four-point bending test setup.

To accommodate the circular cross-section of the pipe, prevent localized indentation (web crippling), and provide adequate bearing areas, custom-machined steel rollers featuring a 15° V-shaped groove were employed for both loading and support points. This geometric adaptation effectively distributed the high contact stresses and mitigated premature local buckling at the load application zones. The testing was continuously recorded until excessive midspan deflection induced physical disengagement between the specimen and the support rollers, a condition operationally defined as the ultimate loss of structural integrity. A schematic representation of the four-point bending test setup is illustrated in Figure 3.

### 3.2. Uniaxial Tensile Test Configuration

Tensile specimens were prepared with an overall length of 393 mm and a designated gauge length of 100 mm. The pinhole defect and the respective repair systems were positioned over the central 57 mm of the gauge zone. The specimen geometry was adapted from the provisions of ASTM E8 [21] and KS B 0802 [22] for tubular cross-sections.

**Figure 4:**
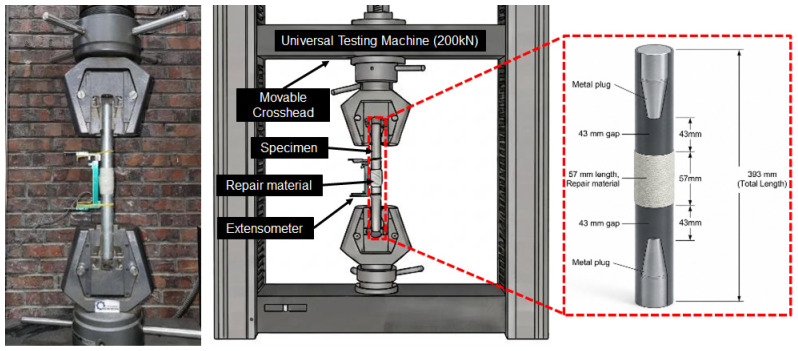
Uniaxial tensile test setup.

A critical mechanical challenge in the uniaxial tensile testing of hollow circular sections is the prevention of cross-sectional ovalization and grip slippage under high axial loads. To circumvent these boundary condition effects, custom-machined solid steel plugs were inserted tightly into the pipe bore at both gripped ends. These structural plugs provided internal radial support, preserving the local section stiffness and completely eliminating wall crushing and relative slip between the specimen and the UTM wedges. A schematic of the tensile test apparatus is presented in Figure 4.

### 3.3. Performance Evaluation Indices

To quantitatively assess and compare the structural recovery across different damage severities and repair techniques, three primary performance indices were established based on the load–displacement responses: (1) Peak Load (P_max_, kN): The maximum load sustained by the specimen prior to the onset of post-peak load degradation, representing the ultimate structural capacity. (2) Displacement at Peak Load (Δ*_max_*, mm): The midspan deflection (bending) or axial elongation (tension) recorded at *P_max_*, providing a measure of deformation capacity. (3) Displacement Ductility Ratio (*µ*): Defined as the ratio of the displacement at peak load to the displacement at the yield point, as follows:

(1) $$\mu =\frac{{∆}_{max}}{{∆}_{y}}$$

where Δ*_max_* is the displacement corresponding to the peak load and Δ*_y_* is the displacement corresponding to the yield load, determined by the 0.2% offset method from the load–displacement curve. A higher ductility ratio indicates a greater capacity for inelastic deformation prior to ultimate failure, which is a critical indicator of structural robustness under dynamic or impact loading conditions (e.g., water hammer, seismic ground motion). The repair efficiency of each technique relative to the unrepaired damaged specimen (H6 or H10) was further quantified as the percentage increase in each performance index:

(2) $$\eta_{P}=\frac{P_{max,\;repair}-P_{max,\;damage}}{P_{max,\;damage}}\times 100\%$$

Analogous expressions were applied to evaluate the recovery efficiency in terms of deformation (*η*_Δ_) and ductility (*η_μ_*). This normalized approach ensures a direct, comparative evaluation of repair effectiveness, irrespective of the absolute inherent strength of the intact pipe.

## 4. Results and Discussion

### 4.1. Four-Point Bending Test Results

#### 4.1.1. Effect of Damage Severity on Bending Performance

**Figure 5:**
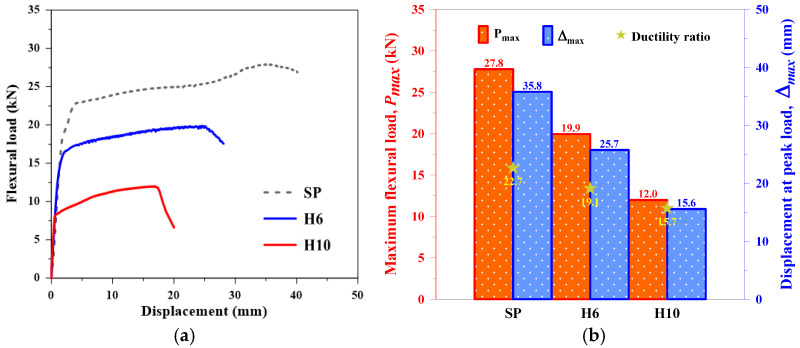
Comparison of flexural performance according to damage severity: (**a**) load–displacement curve; (**b**) comparison of flexural performance metrics.

Prior to evaluating repair effectiveness, the influence of the pinhole damage itself on the bending performance of unrepaired specimens was assessed by comparing the SP (intact control), H6 (6% damage), and H10 (10% damage) specimens. As illustrated in Figure 5, the intact SP specimen exhibited a peak load of 27.8 kN, a displacement at peak load of 35.8 mm, and a displacement ductility ratio (*µ*) of 22.7, reflecting the full ductile flexural capacity of the undamaged KS D 3507 carbon steel pipe. The load–displacement curve of SP displayed a well-defined yield plateau followed by gradual strain hardening, consistent with the ductile behavior expected of low-yield-ratio carbon steel pipe under bending [24].

Introduction of a 6% diameter-ratio pinhole (H6) resulted in reductions of 28.2%, 28.1%, and 15.6% in peak load, displacement at peak load, and ductility ratio, respectively, relative to SP. These reductions are attributable to the localized net-section stress concentration at the pinhole, which accelerates the onset of plastic hinging and curtails post-yield deformation capacity [25]. The more severely damaged H10 specimen demonstrated markedly greater deterioration, with peak load, displacement at peak load, and ductility ratio declining by 56.9%, 56.5%, and 30.7%, respectively, from the intact SP values. The disproportionate performance degradation between the 6% and 10% damage levels suggests a nonlinear relationship between defect size and structural response. As the hole diameter increases, the effective net section decreases while local stress concentration and strain localization become more pronounced, accelerating premature yielding and plastic collapse in thin-walled tubular sections.

#### 4.1.2. Bending Response: 6% Damage Series

**Figure 6:**
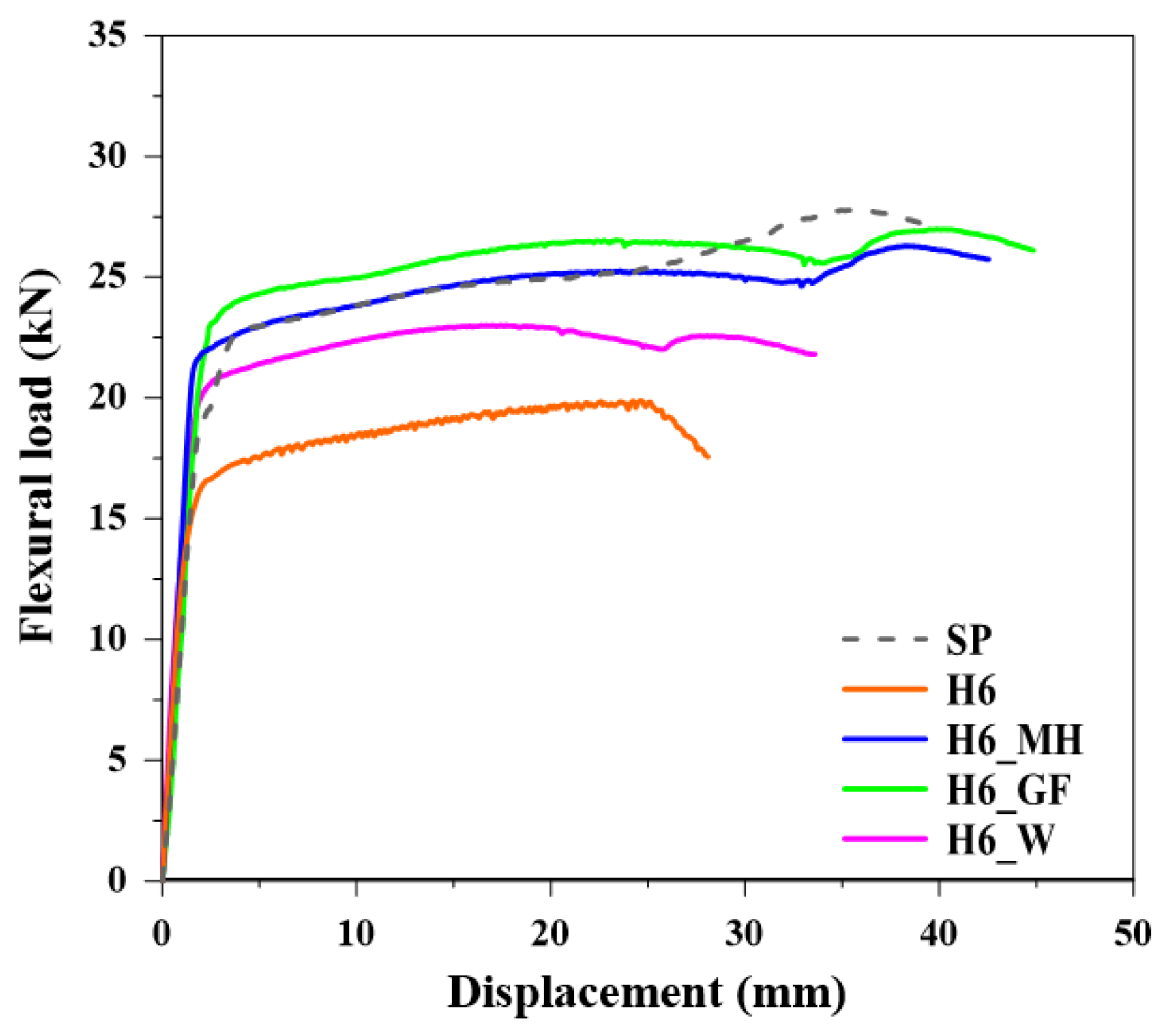
Comparison of flexural performance according to repair method: 6% damage.

The flexural load–displacement curves for all H6-series specimens are presented in Figure 6, with quantitative results summarized in Table 7. All repair techniques applied to the 6% damage specimens produced measurable improvements in all three performance indices relative to the unrepaired H6 baseline. The GFRP composite sleeve (H6_GF) yielded the highest overall repair performance, achieving a peak load of 28.5 kN—essentially recovering the full capacity of the intact SP specimen (102.4% of SP)—along with a 52.7% increase in displacement at peak load and a 35.1% improvement in ductility ratio. The composite sleeve provides circumferential confinement to the damaged region, reducing stress concentration and promoting a more uniform stress distribution along the bonded repair length. As a result, localized yielding and premature fracture initiation at the pinhole boundary are mitigated. This interpretation is consistent with previous studies on FRP-strengthened steel tubular members, which reported reduced stress concentration, delayed crack propagation, and enhanced deformation and energy absorption capacities due to the confinement effect of external composite wrapping [25,26].

The mechanical multi-joint clamp (H6_MH) also demonstrated significant structural recovery, recording a peak load of 26.4 kN (+32.2% over H6), a displacement at peak load of 39.3 mm (+52.6%), and a ductility ratio of 25.1 (+31.1%). The mechanical clamping force successfully suppressed pinhole tearing during bending (see Section 4.1.4), confirming its efficacy as an emergency repair device for minor damage under flexural loading conditions.

The overlay weld (H6_W) produced the most modest improvements among the 6% damage series under bending, with peak load and displacement increasing by only 14.2% and 16.3%, respectively, and the ductility ratio recovering to 21.0 (compared to SP’s 22.7). Despite achieving complete geometric restoration of the damaged section, the overlay weld repair exhibited only moderate recovery in bending capacity and ductility. This behavior may be attributed to the formation of a heat-affected zone (HAZ) surrounding the weld patch. Previous studies have shown that welding-induced thermal cycles can modify the microstructure and local mechanical properties of structural steels, leading to strain localization and reduced deformation capacity in the vicinity of the repaired region [27,28,29].

#### 4.1.3. Bending Response: 10% Damage Series

**Figure 7:**
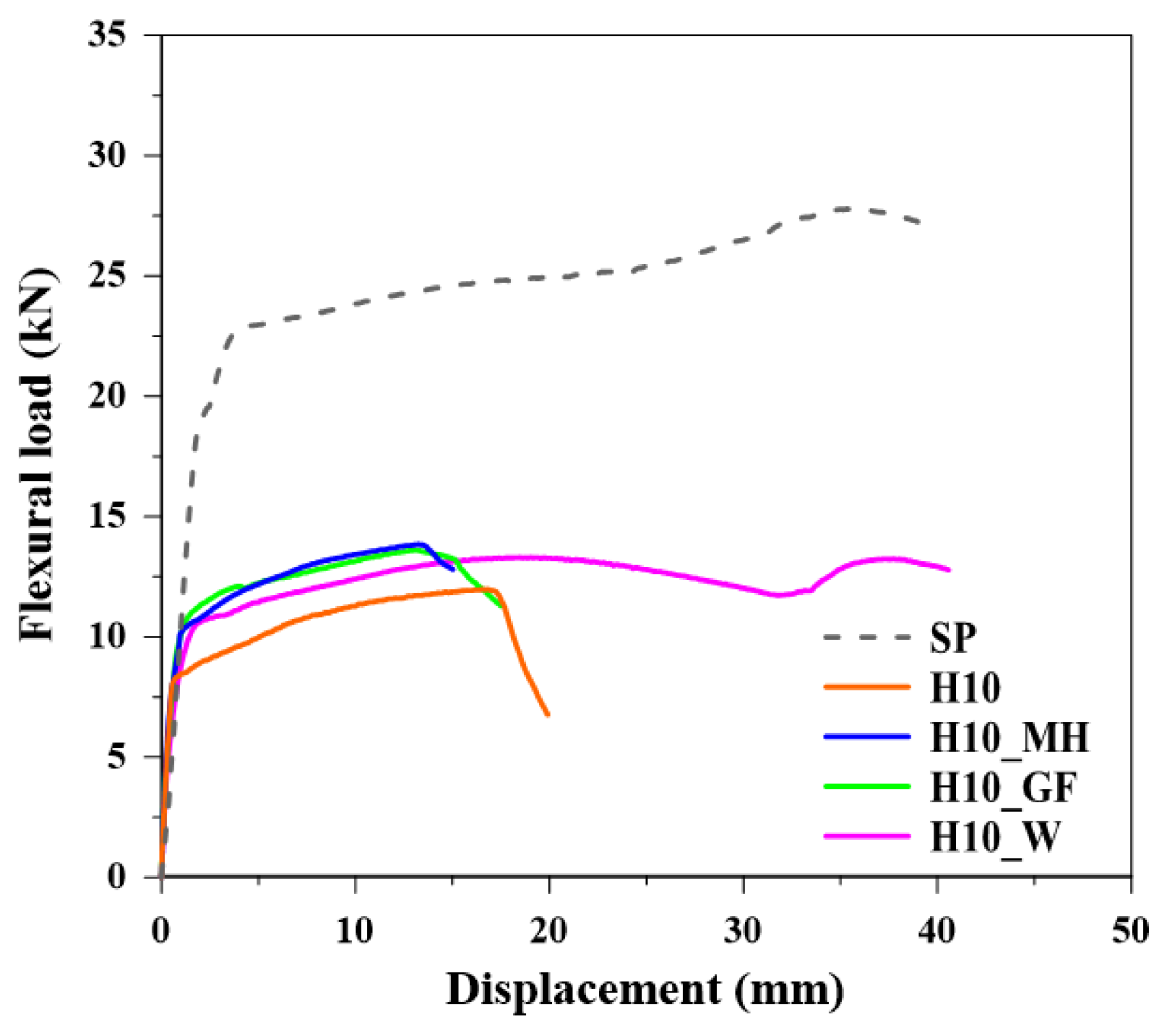
Comparison of flexural performance according to repair method: 10% damage.

The bending performance of the 10% damage series is presented in Table 8 and Figure 7. In contrast to the 6% damage series, the overall repair effectiveness across all methods was generally marginal at this advanced damage level, with the notable exception of the substantial improvements in displacement and ductility achieved by overlay welding.

The multi-joint clamp (H10_MH) yielded only marginal improvements across all performance indices, with peak load, displacement at peak load, and ductility ratio increasing by merely 15.5%, 4.4%, and 3.6%, respectively, relative to the unrepaired H10 baseline. Unlike the 6% damage scenario where the clamp successfully contained the local strain, the enlarged pinhole boundary (10 mm, equivalent to 29.4% of the pipe outer diameter) creates a massive stress riser. The internal strain energy physically overwhelms the external frictional confinement provided by the clamp, allowing premature ductile tearing to propagate well before the section can develop its full plastic moment capacity. The load–displacement curve of H10_MH therefore exhibits a steep post-yield softening branch, confirming that mechanical clamping is structurally insufficient once the damage ratio reaches 10%.

Similarly, the GFRP sleeve (H10_GF) demonstrated critically reduced effectiveness at the advanced damage level. It produced the smallest load increase (+11.1%) and negligible displacement improvement (+3.5%), although its relative ductility recovery (+11.4%) was marginally higher than that of the clamp. This precipitous drop in composite effectiveness is consistent with the mechanics of FRP-reinforced defective sections: as the defect size enlarges, the interfacial shear stress required to redistribute the localized strain grows disproportionately. The single-layer GFRP sleeve applied in this study lacks sufficient radial stiffness to suppress this intensified stress singularity, leading to premature yielding and micro-debonding at the pinhole boundary. This suggests that multi-layer or higher-modulus composite wrapping (e.g., CFRP) is mandatory for addressing advanced damage.

Notably, the overlay weld (H10_W) demonstrated a dramatically different response compared to the other H10 repair techniques, achieving an exceptional 145.4% increase in displacement at peak load and a 70.0% improvement in ductility ratio, with the load–displacement curve closely approaching the profile of the intact SP specimen. This behavior reflects the fundamental difference in repair mechanism: overlay welding physically reconstitutes the full steel cross-section at the defect location, substantially reducing stress concentration rather than merely bridging it externally. As a result, the fracture locus shifts from the pinhole to the base pipe away from the repair zone, restoring near-intact ductile behavior regardless of the initial defect size. This finding is in strong agreement with prior studies on welded patch repairs for corroded steel pipes, which similarly report a transition in fracture location from the defect to the parent material following effective patch welding [16].

From a stress-transfer perspective, the failures of the clamp and GFRP at the 10% damage level highlight the intrinsic limitations of external-confinement strategies. The multi-joint clamp relies exclusively on frictional stress transfer driven by radial compression. However, as the damage size increases, the internal strain energy localized at the defect boundary physically overcomes this frictional limit, resulting in macroscopic ductile tearing prior to full plastic moment development. Similarly, the GFRP system operates via adhesive shear transfer. The intensified stress singularity of a 10 mm defect generates massive interfacial shear stresses that rapidly exceed the adhesive capacity of the single-layer matrix, triggering premature micro-debonding. These failure modes prove that external confinement (frictional or adhesive) is mechanistically insufficient for severe structural defects where direct load-path continuity is absent.

#### 4.1.4. Post-Test Inspection of the Pinhole Geometry and Failure Mode

**Figure 8:**
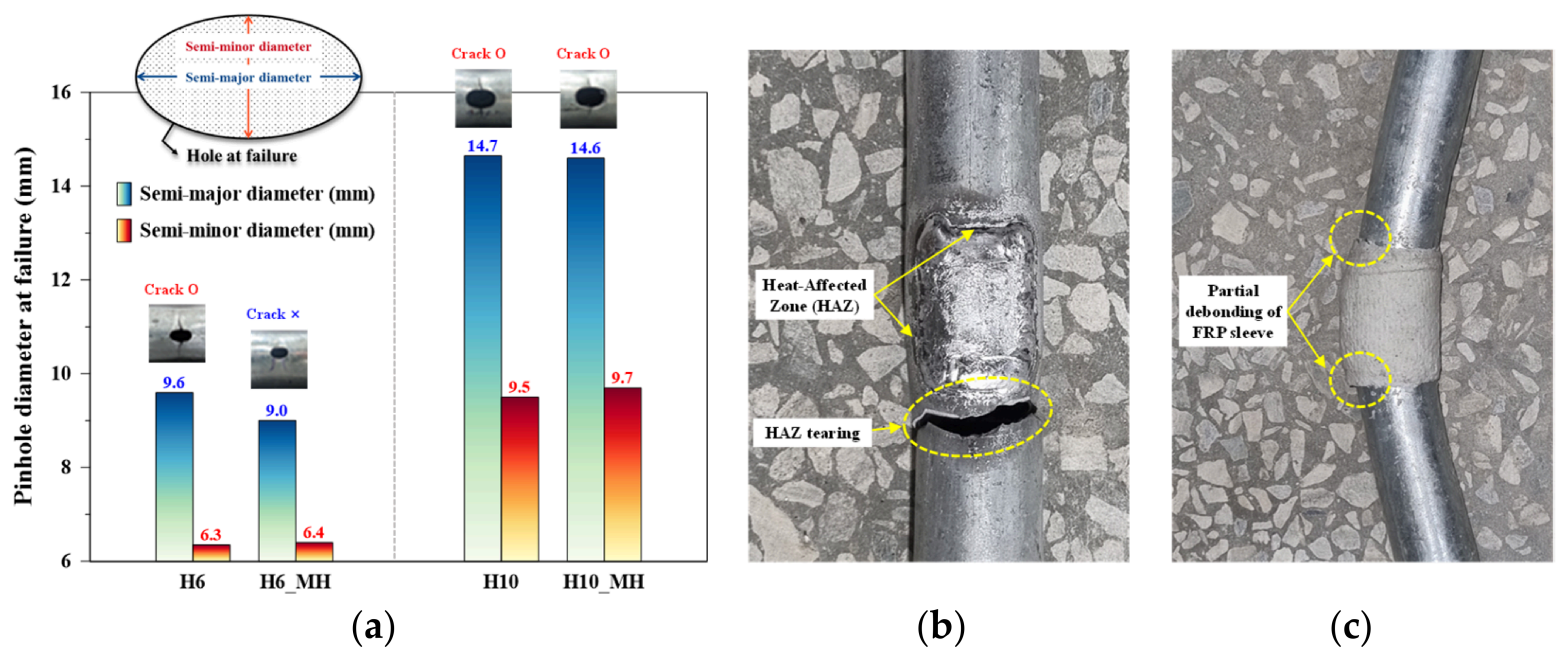
Post-test inspection and failure mode: (**a**) comparison of geometry and failure of unrepaired and multi-joint clamp repaired specimens; (**b**) external inspection of overlay welding specimen (H10_W); (**c**) external inspection of GFRP sleeve specimen (H10_GF).

Post-test inspection of the pinhole geometry confirmed systematically different failure modes depending on both damage severity and repair method (Figure 8). It is noted that post-test inspection was conducted only for the unrepaired cases (H6 and H10) and the multi-joint clamp repairs, as the clamp could be removed after testing to allow direct observation of the defect region without altering the failure characteristics. In contrast, detailed post-test geometric assessment was not feasible for the GFRP sleeve and overlay weld repairs because the repair materials remained permanently bonded or integrated with the pipe surface. Instead, for these specimens, a comprehensive external post-test inspection was performed to characterize their surface deformation, cracking patterns, and interfacial degradation (Figure 8a,b).

In the 6% damage series, the unrepaired H6 specimen exhibited ductile tearing initiating at the pinhole boundary, with the major and minor radii of the deformed hole expanding to 9.6 mm and 6.4 mm, respectively. By contrast, the multi-joint clamp (H6_MH) prevented tearing entirely, with the post-test hole dimensions either remaining unchanged or slightly contracting under the compressive clamping action of the clamp body. This tearing suppression mechanism confirms that for moderate (6%) damage levels, the mechanical clamping force is sufficient to constrain the local strain field below the critical fracture strain of the pipe material. For the 10% damage series, tearing occurred at the pinhole in the clamp-repaired specimen (H10_MH), with post-test major radii expanding significantly. The persistence of pinhole tearing despite the application of the multi-joint clamp at this damage level indicates that the net-section strain at the hole boundary exceeds the clamp confinement capacity, consistent with the limited ductility recovery observed in the load–displacement data. This finding provides a mechanistic basis for the observed performance transition of approximately 6% identified in the experimental data, and aligns with the general principle in pipeline repair engineering that mechanical coupling devices are primarily suited to minor-to-moderate defect containment rather than structural load path restoration [30].

For the welded specimens, external visual inspection (Figure 8b) revealed localized ductile tearing and crack initiation at the boundary of the heat-affected zone (HAZ) directly adjacent to the weld overlay, rather than within the weld metal itself. This indicates that the thermal cycle during welding localized strain and induced localized softening at this metallurgical boundary, rendering the HAZ susceptible to premature fracturing under severe deformation.

For the GFRP sleeve repairs, high flexural loading caused significant pipe curvature (Figure 8c), resulting in a curvature mismatch that led to localized, partial debonding initiating at the outer edges of the GFRP sleeve. This premature debonding limited the full utilization of the composite’s tensile strength before ultimate structural failure. Although only this minor, external debonding was visible during the post-test inspection, a comprehensive synthesis of the mechanical responses in Figure 6 and Figure 7 suggests deeper internal damage. Specifically, for the moderate 6% damage level, the confinement and load-redistribution provided by the GFRP sleeve were sufficient to effectively offset the pinhole defect, yielding robust structural recovery. In contrast, for the more severe 10% damage level, the GFRP’s confinement and load-bridging capacity proved insufficient under the high plastic strain, strongly suggesting that significant, unseen structural tearing or damage progressed internally at the pinhole boundary prior to ultimate failure.

### 4.2. Uniaxial Tensile Test Results

#### 4.2.1. Effect of Damage Severity on Tensile Performance

**Figure 9:**
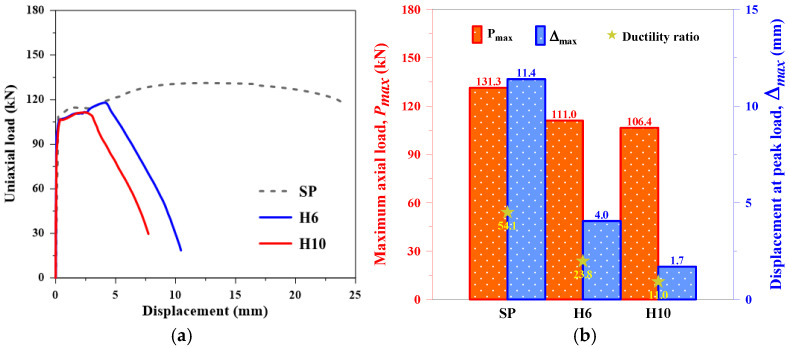
Comparison of tensile performance according to damage severity: (**a**) uniaxial load–displacement curve; (**b**) comparison of tensile performance metrics.

As depicted in Figure 9, the intact SP specimen exhibited a peak tensile load of 131.3 kN, a displacement at peak load of 11.4 mm, and a ductility ratio of 54.1—substantially higher than the bending ductility ratio (22.7), reflecting the inherently greater deformation capacity of the pipe under net-section axial tension compared to bending. This difference arises from the more uniform stress distribution under uniaxial tension, which mobilizes the more uniformly distributed plastic deformation rather than the localized plastic hinging that governs bending failure. The 6% pinhole (H6) reduced peak load, displacement at peak load, and ductility ratio by 15.5%, 64.4%, and 56.0%, respectively, relative to SP. Despite the relatively modest reduction in load capacity, the dramatic decrease in ductility ratio (from 54.1 to 23.8) reveals that even a minor through-wall defect severely impairs the post-yield tensile deformation capacity by acting as a strain localization site. At 10% damage (H10), further reductions of 19.0%, 85.2%, and 79.6% in load, displacement, and ductility were recorded relative to SP, confirming that the 10 mm pinhole creates near-catastrophic loss of tensile ductility—a condition directly relevant to the risk of sudden brittle-like fracture under internal water pressure transients such as water hammer [31].

The disproportionate degradation between the 6% and 10% damage levels profoundly illustrates the extreme sensitivity of thin-walled pipes to geometric discontinuities under axial loading and is primarily attributed to net-section stress concentration. Under uniaxial tension, the pinhole imposes a highly localized reduction in load-bearing area at the net section, and the resulting stress concentration factor governs the onset of plastic strain localization directly at the hole boundary. Because axial tension mobilizes the entire net cross-section in a nominally uniform stress field prior to defect-induced perturbation, even a modest area loss (6%) produces a disproportionately large loss in ductility, as the strain that would otherwise be distributed along the gauge length becomes concentrated at the pinhole edge, triggering premature necking and fracture [32]. Consequently, even a relatively small reduction in cross-sectional area (6%) causes a disproportionately large loss of tensile ductility. As the damage level increases to 10%, the intensified stress concentration further accelerates strain localization, severely limiting global plastic deformation and reducing the ductility ratio to only 20.4% of that of the intact pipe.

#### 4.2.2. Uniaxial Tensile Response: 6% Damage Series

**Figure 10:**
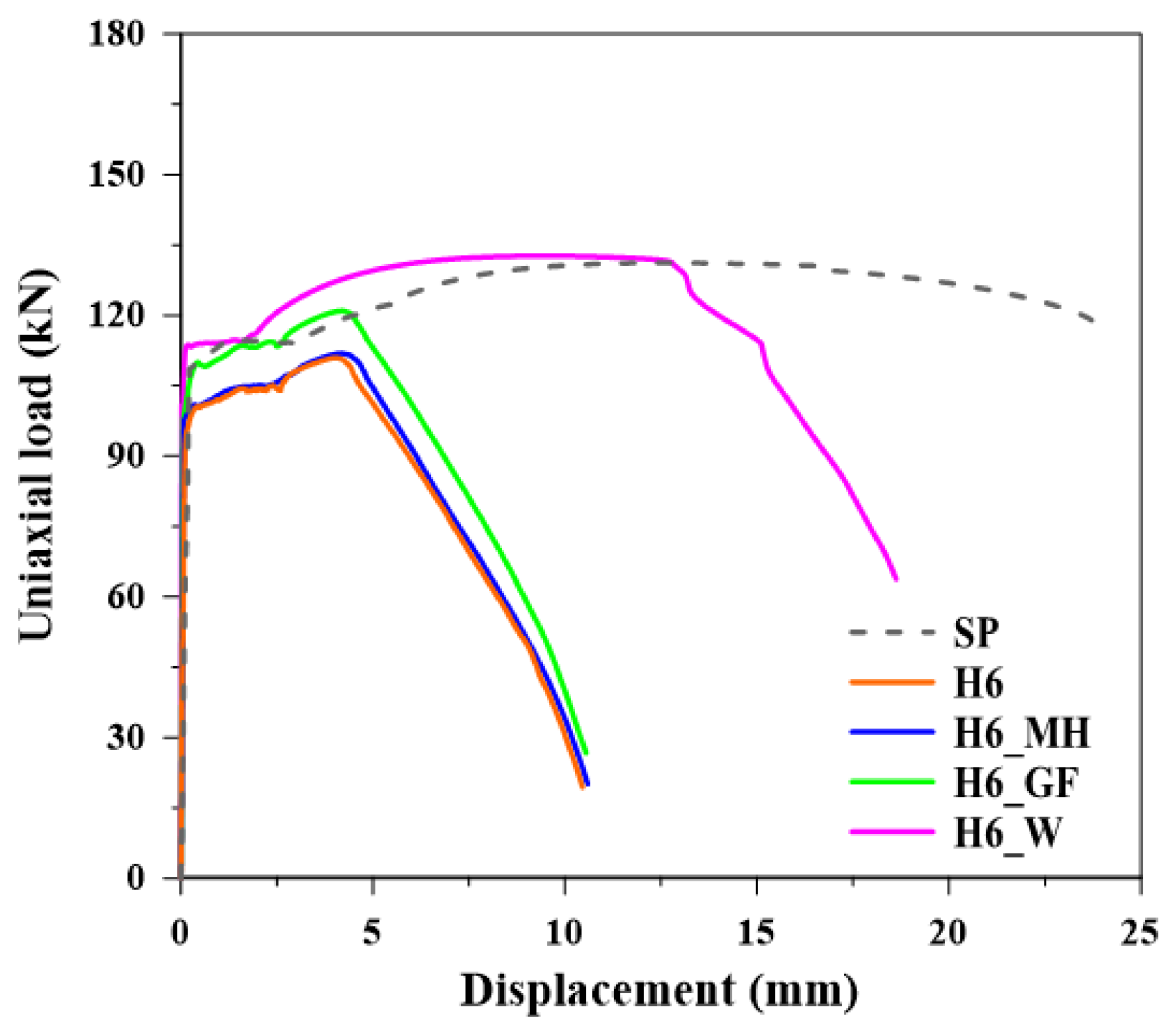
Comparison of tensile performance according to repair method: 6% damage.

A notable contrast emerges between the tensile and bending results for the 6% damage series (Figure 10 and Table 9). While the multi-joint clamp and the GFRP sleeve produced substantial improvements under bending, their effectiveness under direct tension was markedly more limited. Specifically, the load–displacement response of the Hinge-type clamp (H6_MH) closely followed that of the unrepaired H6 baseline, providing negligible structural enhancement under axial tension. The GFRP sleeve (H6_GF) offered a marginal improvement in peak load capacity, but its displacement and ductility enhancements remained similarly minimal. This suggests that under uniaxial tension, the external mechanical confinement provided by the clamp and the composite sleeve is insufficient to significantly redistribute the severe localized strain concentration at the pinhole boundary when the loading direction acts parallel to the defect plane [33]. In this loading configuration, all tensile force must be transmitted through the net section adjacent to the hole, regardless of the repair mechanism, unless the repair material is capable of directly carrying a portion of the axial load.

By contrast, the overlay weld (H6_W) achieved a peak load of 129.3 kN (98.5% of intact SP), with displacement at peak load and ductility ratio recovering to 9.4 mm and 45.3, respectively—corresponding to remarkable increases of 131.4% and 90.2% over the H6 baseline. The weld repair’s exceptional tensile performance stems from the fact that the patch plate directly augments the net cross-sectional area at the defect location, restoring the full load path and eliminating the stress concentration [34]. Post-test observation confirmed that fracture in H6_W occurred in the base pipe away from the welded patch, indicating that the repaired section had exceeded the strength of the surrounding parent material—the defining criterion for a structurally complete repair.

The stark contrast in tensile recovery clearly demonstrates how repair mechanisms interact with external loads. Under pure uniaxial tension, the applied force acts parallel to the defect plane. In this loading regime, the clamp (frictional confinement) and GFRP (adhesive confinement) fail to establish a direct and continuous axial load-transfer path. Consequently, longitudinal stresses are redirected around the repair region and concentrated at the reduced steel net-section, triggering localized plastic deformation and premature failure. Conversely, overlay welding provides direct metallurgical bonding and effective stress transfer. By physically fusing a matching steel patch across the defect, it restores the continuity of the steel section. This bonding mechanism significantly reduces stress concentration and shifts the failure location into the parent pipe material, explaining its superior tensile ductility recovery.

#### 4.2.3. Uniaxial Tensile Response: 10% Damage Series

**Figure 11:**
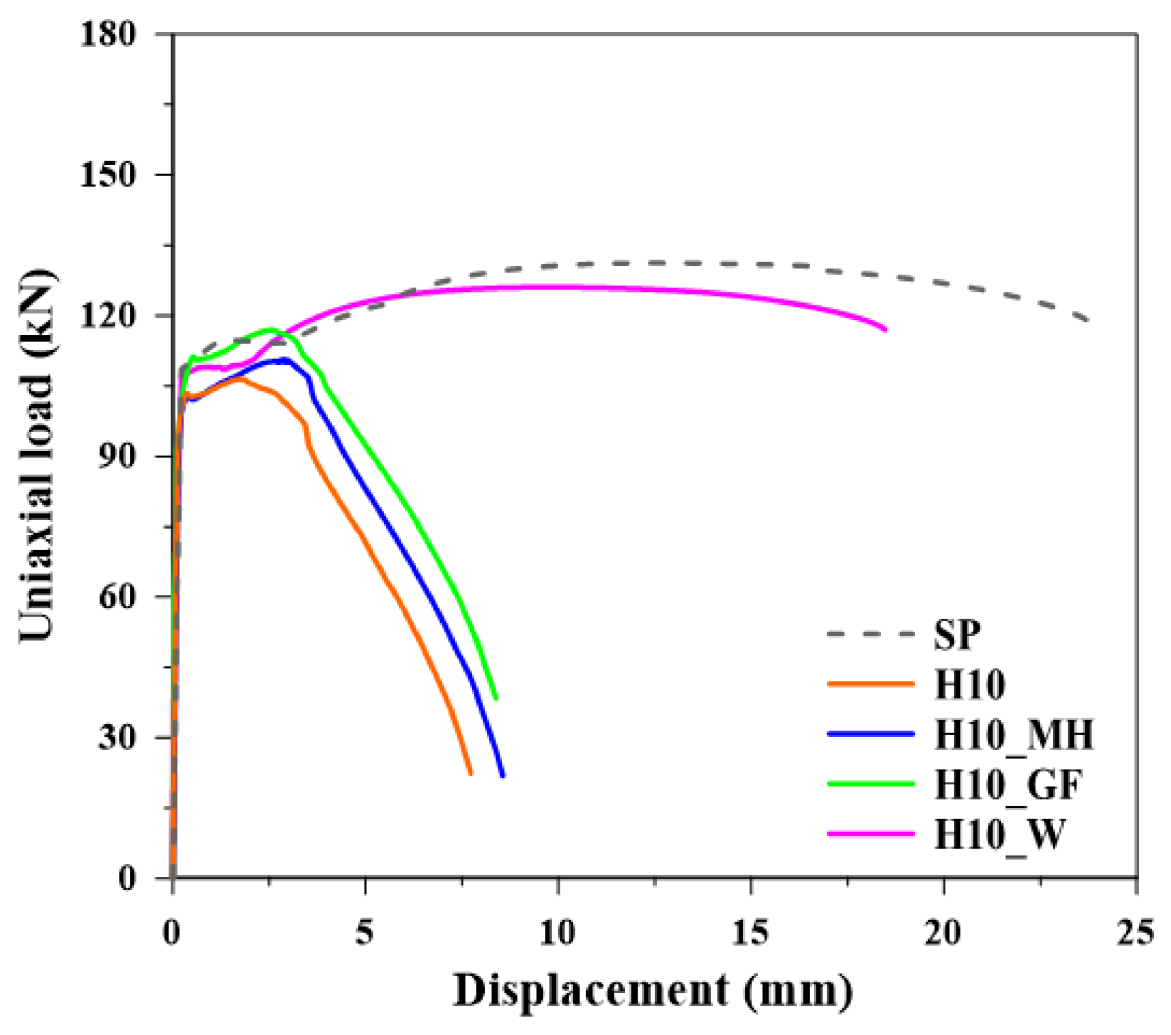
Comparison of tensile performance according to repair method: 10% damage.

As illustrated in Figure 11 and Table 10, the tensile results for the 10% damage series further substantiate the distinction between load-path-restoring and externally confining repair strategies. The multi-joint clamp (H10_MH) and GFRP sleeve (H10_GF) achieved only modest peak load gains over H10, with displacement at peak load remaining critically low. Despite these limited absolute improvements, the relative increase in ductility ratio for H10_MH (+46.5%) appears substantial; however, the absolute ductility ratio value (16.2) remains far below the intact SP level (54.1), indicating that meaningful structural ductility has not been restored.

The critical finding in this series is the outstanding performance of H10_W, which achieved a peak load of 126.1 kN (+18.5% over H10), a displacement of 9.3 mm (+449.1%), and a ductility ratio of 45.4 (+311.9%)—approaching the intact SP values more closely than any other repair–damage combination in the study. The fracture in H10_W again occurred in the parent pipe away from the weld, confirming complete structural restoration at the repair location. The superior performance of overlay welding under tension, irrespective of damage severity, is consistent with findings in analogous studies on patch-welded corroded pipe sections, where cross-sectional area restoration has been identified as the primary determinant of tensile performance recovery [35].

### 4.3. Comparison and Practical Implications: Bending vs. Tension Performance

**Figure 12:**
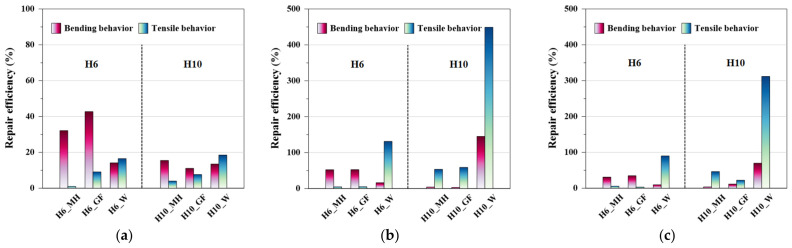
Quantitative evaluation of repair effects in bending and tensile behavior: (**a**) peak load; (**b**) displacement at peak load; (**c**) ductility ratio.

**Figure 13:**
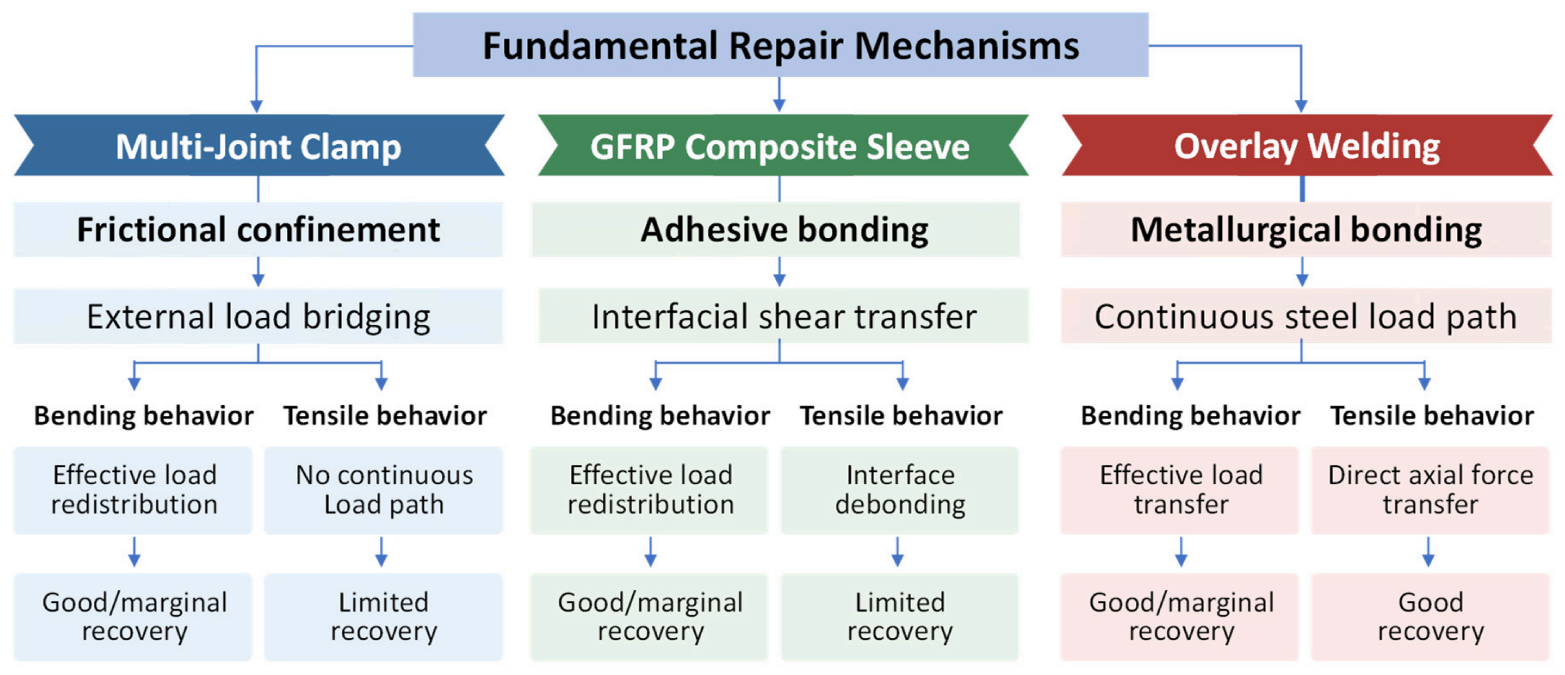
Schematic summary of the three repair mechanisms and their differential effectiveness under bending versus tensile loading.

A holistic synthesis of the experimental results indicates that the asymmetric structural recovery observed in this study (see Figure 12) is primarily governed by the stress-transfer mechanism of each repair system. To further facilitate interpretation of these mechanisms, Figure 13 presents a conceptual illustration summarizing the three fundamental repair strategies—frictional, adhesive, and metallurgical—and their respective responses under bending and axial tensile loading. Non-metallurgical techniques (multi-joint clamp and GFRP sleeve) function as external load-bridging systems, transferring stresses through frictional confinement and adhesive interfacial shear without restoring a continuous load path across the defect [36,37].

Under bending, these mechanisms effectively redistributed stresses and provided local confinement, resulting in substantial recovery of structural capacity. However, under axial tension, neither repair system was able to fully compensate for the loss of the load-bearing cross-section, leading to limited tensile-strength recovery despite their favorable flexural performance. In contrast, overlay welding restores the damaged cross-section through metallurgical fusion, thereby re-establishing a continuous load path across the defect. This repair mechanism consistently achieved superior structural recovery under both bending and tensile loading, indicating that direct restoration of load continuity is fundamentally more effective than external confinement for recovering tensile resistance. The slightly reduced bending performance observed in the welded specimens at the 10% damage level may be attributed to localized softening and stiffness mismatch within the heat-affected zone (HAZ), as reported in previous welding studies [38].

From an engineering perspective, these findings demonstrate that repair performance is governed not only by damage severity but also by the interaction between the repair mechanism and the applied loading condition. Agricultural pipelines in service are commonly subjected to combined loading, including longitudinal axial tension induced by ground settlement or thermal contraction together with externally induced bending. Consequently, repair methods selected solely on the basis of leakage control or flexural performance may not provide sufficient resistance under tensile loading. Therefore, repair selection should explicitly consider the dominant in-service stress state, while future studies should further validate these mechanisms under cyclic, fatigue, and dynamic loading conditions to establish comprehensive performance-based rehabilitation guidelines.

## 5. Conclusions

This study comprehensively investigated the structural recovery and fundamentally distinct stress-transfer mechanisms of three representative retrofitting techniques applied to thin-walled agricultural steel pipelines. Pinhole defects, simulating varying severities of localized pitting corrosion (6% and 10% diameter-to-circumference ratios), were evaluated under both pure bending and uniaxial tensile stress states. Based on the rigorous experimental results and failure mode analyses, the following principal conclusions are drawn:

(1) Damage severity significantly influenced the structural performance of corroded steel pipelines under both bending and tensile loading. Structural degradation increased nonlinearly with increasing pinhole size, and the 10% damage condition resulted in severe reductions in strength and ductility, particularly under tensile loading. These results indicate that heavily corroded pipelines are highly susceptible to fracture under pressure-induced tensile stresses.
(2) An apparent transition in performance was observed at approximately 6% damage regarding the applicability of external-confinement repair methods. Within the investigated range, the repair techniques generally maintained structural performance and ductile behavior at this damage level. However, their effectiveness decreased substantially at the 10% damage level, indicating that more severe defects may exceed the effective confinement capability of externally applied repair systems.
(3) The structural effectiveness of the repair techniques was found to depend strongly on the applied loading condition. Adhesive (GFRP) and frictional (Clamp) repair systems provided substantial flexural recovery by bridging the defect under bending, indicating that these techniques are particularly effective when bending is the dominant loading condition. Specifically, for the 6% damage level, the GFRP sleeve significantly restored the bending capacity, whereas its improvement in tensile ductility remained minimal. This limited contribution to axial load transfer and tensile ductility is attributed to the fact that neither repair method restores a continuous metallic load path across the defect.
(4) Overlay welding demonstrated the most reliable overall structural recovery among the three repair techniques. By reconstituting the net cross-sectional area at the defect, welding effectively neutralized the stress concentration and shifted the fracture locus from the pinhole to the parent pipe material. The extent of recovery, however, depended on the loading mode and the performance criterion. While ductility indices were largely restored under tensile loading, peak load recovery under bending remained partial, particularly at the 10% damage level (H10_W: 13.6 kN vs. SP: 27.8 kN). Therefore, the effectiveness of overlay welding should be interpreted in the context of the specific loading condition and performance criterion.
(5) The profound disparity in performance under bending versus tension reveals a critical vulnerability in applying external-confinement repairs to highly pressurized pipelines based solely on empirical or flexural data. This underscores the urgent necessity to transition from current prescriptive maintenance guidelines (e.g., KDS 67 25 90:2018) to load-specific, performance-based engineering criteria.

The empirical evidence and mechanistic insights derived from this study provide a robust foundation for establishing quantitative repair selection criteria for aging agricultural infrastructure. Since the present investigation was limited to quasi-static bending and uniaxial tensile tests, the reported repair effectiveness should be interpreted within these loading conditions. Future research should prioritize full-scale validation on large-diameter pipelines and multi-axial combined loading conditions (simultaneous internal pressure and external bending), as well as cyclic, fatigue, and dynamic loading conditions, together with the long-term environmental durability of interfacial bond mechanisms.

## Figures and Tables

**Table 1:** Mechanical properties of steel pipe material (KS D 3507, 25A).

Property	KS Requirement	Mill Certificate	Measured (Avg.)
Yield strength (MPa)	≥200	221	365 (5.6)
Tensile strength (MPa)	≥340	374	418 (6.3)
Elongation (%)	≥30	43	50 (0.2)

(): standard deviation.

**Table 2:** Chemical composition of steel pipe material (weight %).

	C	Si	Mn	P	S
	×100 wt.%		×1000 wt.%		
KS D 3507	≤28	≤35	≤80	≤4	≤4
Measured	7.98	1	41	7	6

**Table 3:** Multi-joint clamp properties and geometry.

Parameter	Value
Outer diameter compatibility (mm)	33.4–34.6
Clamp length (mm)	57
Allowable expansion (mm)	32
Working pressure (MPa)	2.8
Unit weight (kg)	0.32
Tightening torque (N·m)	3–5

**Table 4:** Properties of FRP composite sleeve.

Parameter	Value
Flexural modulus of elasticity (GPa)	6.14 (at 0 °C), 4.57 (at 90 °C)
Glass fiber content (%)	46–55
Polyurethane resin content (%)	45–54
Tensile strength (MPa)	182 (at 23 °C), 214 (at −60 °C), 245 (at −160 °C)
Thermal resistance (°C)	200
Shore hardness	76
Charpy impact strength (kJ/m^2^)	29

**Table 5:** Experimental matrix for the small-diameter steel pipe specimens.

Specimen	Pinhole Damage	Repair method	Description
SP (Steel Pipe)	None	None	Intact control specimen
H6	6% (*ϕ*6.5 mm)	None	Damaged, unrepaired
H6_MH		Multi-joint (Hinge type)	Damaged, clamp (hinge) repair
H6_GF		GFRP composite sleeve	Damaged, GFRP repair
H6_W		Overlay welding	Damaged, patch welding repair
H10	10% (*ϕ*10 mm)	None	Damaged, unrepaired
H10_MH		Multi-joint (Hinge type)	Damaged, clamp (hinge) repair
H10_GF		GFRP composite sleeve	Damaged, GFRP repair
H10_W		Overlay welding	Damaged, patch welding repair

**Table 6:** Core mechanisms and primary failure modes of repair techniques.

Repair Technique	Core Mechanism	Load Transfer	Anticipated Failure Modes
Multi-joint clamp (MH)	Mechanical bridging & clamping	Friction/bearing resistance via mechanical fastening	Bolt yielding/Loosening Interfacial slippage Localized buckling at clamp edges
GFRP composite sleeve (GF)	Composite wrap confinement	Interfacial shear via adhesive layer	Delamination/Debonding Interlaminar matrix cracking FRP composite rupture
Overlay welding (W)	Metallurgical bonding & sealing	Direct shear/tension across the weld bead	HAZ cracking Weld metal fracture Base metal yielding

**Table 7:** Four-point bending test results: 6% damage.

Specimen	*P_max_* (kN)	Δ*_max_* (mm)	*µ*	*η_P_* (%)	*η*_Δ_ (%)	*η_µ_* (%)
SP	27.8 (1.13)	35.8 (3.30)	22.7 (3.97)	-	-	-
H6	19.9 (0.94)	25.7 (3.30)	19.1 (0.14)	-	-	-
H6_MH	26.4 (0.31)	39.3 (0.49)	25.1 (0.37)	32.2	52.6	31.1
H6_GF	28.5 (0.46)	39.3 (0.59)	25.8 (2.30)	42.7	52.7	35.1
H6_W	22.8 (1.06)	29.9 (4.11)	21.0 (1.90)	14.2	16.3	9.6

*η_P_*, *η*_Δ_, *η_µ_*: percentage increase relative to H6 baseline; (): standard deviation.

**Table 8:** Four-point bending test results: 10% damage.

Specimen	*P_max_* (kN)	Δ*_max_* (mm)	*µ*	*η_P_* (%)	*η*_Δ_ (%)	*η_µ_* (%)
SP	27.8 (1.13)	35.8 (3.30)	22.7 (3.97)	-	-	-
H10	12.0 (4.72)	15.6 (2.36)	15.7 (3.77)	-	-	-
H10_MH	13.8 (4.16)	16.3 (4.83)	16.3 (3.54)	15.5	4.4	3.6
H10_GF	13.3 (3.19)	16.1 (3.29)	17.5 (2.54)	11.1	3.5	11.4
H10_W	13.6 (1.28)	38.2 (1.51)	26.7 (3.36)	13.5	145.4	70.0

*η_P_*, *η*_Δ_, *η_µ_*: percentage increase relative to H10 baseline; (): standard deviation.

**Table 9:** Tensile test results: 6% damage.

Specimen	*P_max_* (kN)	Δ*_max_* (mm)	*µ*	*η_P_* (%)	*η*_Δ_ (%)	*η_µ_* (%)
SP	131.3 (1.77)	11.4 (1.21)	54.1 (0.16)	-	-	-
H6	111.0 (3.94)	4.1 (0.02)	23.8 (2.24)	-	-	-
H6_MH	112.1 (0.44)	4.3 (0.15)	24.5 (0.57)	1.0	4.9	3.0
H6_GF	121.1 (1.70)	4.3 (0.12)	24.5 (1.81)	9.1	5.2	3.0
H6_W	129.3 (2.42)	9.4 (0.35)	45.3 (3.93)	16.5	131.4	90.2

*η_P_*, *η*_Δ_, *η_µ_*: percentage increase relative to H6 baseline; (): standard deviation.

**Table 10:** Tensile test results: 10% damage.

Specimen	*P_max_* (kN)	Δ*_max_* (mm)	*µ*	*η_P_* (%)	*η*_Δ_ (%)	*η_µ_* (%)
SP	131.3 (1.77)	11.4 (1.21)	54.1 (0.16)	-	-	-
H10	106.4 (3.15)	1.7 (0.49)	11.0 (2.19)	-	-	-
H10_MH	110.7 (2.62)	2.6 (0.29)	16.2 (1.21)	4.0	53.9	46.5
H10_GF	114.6 (2.60)	2.7 (0.17)	13.5 (1.32)	7.7	59.2	22.5
H10_W	126.1 (1.17)	9.3 (0.56)	45.4 (1.55)	18.5	449.1	311.9

*η_P_*, *η*_Δ_, *η_µ_*: percentage increase relative to H10 baseline; (): standard deviation.

## Data Availability

The data presented in this study are available upon request from the corresponding author due to a planned future patent application and the requirement for prior review and approval by the funding agency before public disclosure.
